# Attitudes and Knowledge of Israeli Ultra-Orthodox and Religious Jewish Nursing Students Toward the Use of Medical Cannabis

**DOI:** 10.1007/s10943-025-02381-9

**Published:** 2025-07-07

**Authors:** Gregory Kozlov, Oren Wacht, Orli Grinstein-Cohen

**Affiliations:** 1 Department of Nursing, Faculty of Health Sciences, Leon and Matilda Recanati School for Community Health Professions, Ben-Gurion University of the Negev, 1 David Ben Gurion Boulevard, Beer Sheva, Israel; 2 Department of Emergency Medicine, Faculty of Health Sciences, Leon and Matilda Recanati School for Community Health Professions, Ben-Gurion University of the Negev, 1 David Ben Gurion Boulevard, Beer Sheva, Israel

**Keywords:** Medical cannabis, Nursing students, Attitudes, Knowledge, Rabbi

## Abstract

There has been a sharp increase in recent years in the use of medical cannabis in Israel. However, little is known about the attitudes and knowledge about medical cannabis among nursing students, particularly those from the religious/ultra-Orthodox sector. It is important for nurses to know about medical cannabis because they are at the forefront of care and provide patient guidance. A cross-sectional study sampled 221 religious/ultra-Orthodox nursing students who were studying for a BN or MN degree. Online questionnaires asked for demographic details and tested their attitudes, beliefs, and knowledge regarding the use of medical cannabis. Data analysis was conducted using the Pearson Correlation, *t* tests for independent samples, unifactorial and multiple regression variance tests to predict positive attitudes toward medical cannabis. The findings show that the attitudes of nursing students from the religious/ultra-Orthodox sector regarding the use of medical cannabis are more negative than those of other nursing students. Participants who had been exposed to medical cannabis expressed more positive attitudes. However, most participants oppose cannabis being made legal for recreational use, believe it is addictive, and show a very low medical cannabis usage rate. A negative connection was also found between degree of religiosity and attitudes toward medical cannabis. The knowledge of religious/ultra-Orthodox nursing students regarding medical cannabis was found to be low, and they supported its inclusion in the curriculum. The findings indicate a need to include medical cannabis in the academic curriculum to increase knowledge about it among healthcare professionals. The research should be extended to a representative sample of additional populations and compare between staff who studied about medical cannabis and those who did not. Depth interviews should be conducted with policy makers to discover what has been done and will be done to train the next generation of students.

## Introduction and Literature Review

### Cannabis

The healing properties of cannabis have been known since about 2800 BCE, and medical use existed already at that time in Chinese medicine and in ancient Egypt (Gottkind, 2014). The use of the plant was extensive and recommended by physicians. However, over the years, due to non-scientific political and social reasons, the use of the cannabis plant became illegal (Baron, 2015).

The varied use of the cannabis plant for both non-medical and medical purposes was common up to 1937. After the prohibition of alcohol was lifted, American authorities condemned the use of cannabis, blaming it for insanity, moral and intellectual decline, violence, and various crimes. In this year, cannabis was defined as a drug in the USA, its consumption was banned, and it was made illegal.

Over the years, cannabis was defined as a dangerous drug in most countries and according to United Nations conventions. However, the use of the drug for medical purposes is now becoming very popular. Research into cannabis is expanding, and its medical possibilities are being revealed (Ben Amar, 2006; Timna, 2016).

### Medical Cannabis

Many patients suffering from various diseases report an improvement in their condition following treatment with cannabis. As a result of these reports, many ministries of health around the world, including Israel, currently approve the consumption of cannabis for medical purposes (Timna, 2016).

Medical cannabis is prescribed to help many patients: to improve appetite among patients with AIDS; to treat nausea and vomiting among cancer patients receiving chemotherapy; to relieve muscle contractions among multiple sclerosis patients; and to help inflammatory bowel disease patients (Ben Amar, 2006; Timna, 2016).

Cannabinoids also have the potential to treat chronic pain, Tourette syndrome, epilepsy, and glaucoma (Pedersen & Sandberg, 2013). In addition, from 2016 there have been growing reports in the literature indicating a beneficial effect of cannabinoids on post-traumatic stress disorder (PTSD) (Timna, 2016).

### Medical Cannabis in Israel

As of 2019, Israel has approved the use of medical cannabis for PTSD and eight other medical conditions: chronic pain, muscular sclerosis, Chron's, Parkinsons, epilepsy, cancer, terminal patients, and AIDS patients (Landshaft et al., 2019). As of January 2022, two additional conditions have been added: behavioral issues related to the autistic spectrum and behavioral issues caused by dementia (Ministry of Health, 2022).

Since the approval of medical cannabis in Israel, the number of patients has been rising sharply (Avisar, 2019; Timna, 2016). At present, about 111,000 patients in Israel have received a permit to consume cannabis (Medical Cannabis Unit, 2022), and the numbers are still growing. Therefore, it is important for the care team in general and for nurses in particular to possess reliable information about medical cannabis. The team should be familiar with its advantages, disadvantages, indications, and treatment methods.

### Nurses and Medical Cannabis

According to Gottkind (2014), the nurses are those who accompany the patients and their families around the clock. Today there are increasing numbers of patients and families interested in the treatment options, asking questions about them, and expecting the nursing staff to provide clear and unambiguous answers. The families see nurses as a reliable, efficient, and available source of information, and thus the nursing staff is required to provide explanations and guidance in this area.

Studies conducted with healthcare students have shown that despite the increasing usage of medical cannabis, the students have very little knowledge about it. This makes them feel uncomfortable and insecure when working with patients. It transpires that academic institutions do not train students about medical cannabis (Caligiuri et al., 2018; Evanoff et al., 2017; Pereira et al., 2020; Zolotov et al., 2021a, 2021b). Therefore, most students supported receiving training in this area and including the topic of medical cannabis in the academic curriculum (Pereira et al., 2020).

### Attitudes to Medical Cannabis

Despite the growing support for cannabis as a medical treatment, not enough is known about the attitudes of healthcare students toward its use. According to Chan and colleagues (2017), earlier studies about physicians' attitudes found that physicians who were less religious and more liberal were associated with greater support of the legalization of cannabis, which was linked to "immorality" and "permissiveness". In addition, those who had used cannabis in the past showed more positive attitudes toward medical cannabis (Chan et al., 2017).

The use of cannabis is permitted in Israel only for certain medical uses and it is still controversial and raises public and professional debates. The few studies published about the attitudes of physicians toward medical cannabis found that most of them do not support it (Ebert et al., 2015). Therefore, it was important to investigate the attitudes of nursing students, especially because they are in direct contact with patients and need to provide professional responses to their patients.

### Objective

This study examined the attitudes of nursing students from the religious/ultra-Orthodox sector. Ultra-Orthodox students come from a closed sector that is different from the secular sector. Bilu and Witztum (1993) describe the ultra-Orthodox lifestyle as drastically different and contrasting to the modern, non-ultra-Orthodox population.

If we compare the research population to medical students in a study conducted in Russia, we can see that the research population has more positive attitudes compared with students in Russia, who do not support the use of medical cannabis. The difference is probably related to cannabis use for both recreational and medical use being banned in Russia (Gritsenko et al., 2020). Therefore, we can assume that the attitudes of religious and ultra-Orthodox nursing students in Israel regarding the use of medical cannabis will differ from those of the secular population. The ultra-Orthodox community may face stigma related to mental health and substance use, which can influence attitudes toward medical cannabis (Gabbay et al., 2017).

This study was conducted following the study by Isralowitz and colleagues (2021), which examined mainly secular students.

## Research Hypothesis

The attitudes of nursing students from the religious / ultra-Orthodox community regarding medical cannabis will be found to be more negative compared with other populations examined in the literature and a negative association will be found between the level of religiosity and attitudes toward medical cannabis. The more religious the student, the more negative their attitudes toward medical cannabis.

## Methods

### Setting and Procedures

The study is a cross-sectional study conducted in May–June 2020 (second semester). The data were collected from nursing students at the Lev Academic Center who were studying for B.N. and M.N. degrees in all branches of Lev Academic Center. This is a public academic religious college that has the largest nursing department in Israel.

During the academic year in question, the nursing department contained 1,050 students. The college includes Jewish studies, and there are separate campuses for men and women. The sample includes 221 students who answered all the questions in the questionnaire, comprising about 21.5% of all the nursing students that year.

All the nursing students received a link to an online questionnaire by email or class WhatsApp groups, with an explanation message. In order to maintain the respondents' anonymity, the researcher was not a member of the WhatsApp group. The questionnaire was also sent to the WhatsApp group and the students' email address, especially for ultra-Orthodox students who did not use WhatsApp and/or Facebook. The questionnaire was completely anonymous. No names, ID numbers, or telephone numbers were collected, and therefore it is impossible to identify the respondents. The link sent to the students clarified that participation is completely voluntary. Clicking on the link led to an explanation about the research and a consent form to participate in it.

Students were able to stop answering at any stage or to choose not to answer some of the questions. The students were requested to answer an anonymous questionnaire examining attitudes, beliefs, and knowledge regarding the use of medical cannabis, and at the end a section of demographic data. The questionnaire was identical for all the nursing programs included in the study. No incentive was granted for participation.

The request to conduct the study was approved by the Faculty Research Ethics Committee of the Life and Health Sciences Faculty, Lev Academic Center (no number available).

#### Data Analysis

The demographic data were described using descriptive statistics. To examine the hypotheses, the data were analyzed using the t-test and the one-way analysis of variance (ANOVA) test. All the statistical data analysis was conducted using IBM SPSS statistics version 29.

The sample size was calculated using OpenEpi software for calculating sample size in proportion to the frequency of the variable in the population. The calculation included the anticipated frequency of 78.1%, a population of 1,050 students, a design effect = 1.0, and the calculation had a confidence level of 95%. The results showed the need for at least 211 participants to achieve statistical significance. Assuming a response rate of 75%, a sample of 282 participants would be required. The questionnaire was sent to all the nursing students at Lev Academic Center in the second semester of 2020–21.

### Measurements

The questionnaire was based on the Medical Marijuana Questionnaire (MMQ), developed by Chan and colleagues (Chan et al., 2017). After receiving permission, the questionnaire was culturally adapted, translated (using the back-and-forth method) into Hebrew, and validated. The questionnaire included 23 questions. First, 10 questions dealing with attitudes, then one question (with 18 items) examining perceived the efficacy of cannabis, then five questions testing knowledge, and finally we used seven questions about demographics. This questionnaire has already been used in several studies around the world.

The demographic information included age, gender, religiosity level, personal and occupational status, education level, and work experience. Participants were queried about the frequency of their own personal use of cannabis for medical and/or recreational purposes (from “Never” to “Daily”), and were additionally asked if they have a friend and/or family member who use or had used cannabis for medical and/or recreational purposes (yes/no).

The questionnaire additionally included the following sections:

*Attitudes and beliefs*. Sample items are “Physicians should recommend cannabis as a medical therapy”, and “There are significant mental health benefits using medical cannabis”. Possible answers were on a six-point Likert scale (from strongly agree to strongly disagree). For analytic purposes, answers were collapsed into a dichotomous variable of “Agree” (strongly agree, agree, and somewhat agree) versus “Disagree” (strongly disagree, disagree, and somewhat disagree).

*Perceived efficacy of cannabis.* Participants were asked to rate their perceived efficacy of cannabis for 18 different medical conditions, on a six-point Likert scale (from very effective to very ineffective). The list of medical conditions was compiled from ailments that have scientific and/or regulatory acceptance as qualifying conditions for medical cannabis use, for example cachexia, fibromyalgia, multiple sclerosis, and epilepsy. In the end, we only examined the participants' answers regarding nine medical conditions from the list, those for which there is a recognized treatment indication for medical cannabis based on the Israeli Ministry of Health's Circular from 2019 (Landshaft et al., 2019).

For analytic purposes, answers were collapsed into a dichotomous variable of “Effective” (strongly effective, effective, and somewhat effective) versus “Ineffective” (strongly ineffective, ineffective, and somewhat ineffective). The results present the participants' answers only regarding nine of the medical conditions from the list, those recognized as medical indications for the use of medical cannabis according to the Israeli Ministry of Health Circular from 2019 (Landscaft et al., 2019).

*Knowledge and training.* Participants were asked if they feel prepared to answer questions from patients about medical cannabis, and whether they think that students in their professional field should receive formal education about cannabis. We additionally asked participants if they had previously received formal education about medical cannabis in class and/or in a clinical practice setting, if they believed that relevant training should be provided within academic curriculum and field practice requirements, and if such training should be mandatory to professionals who recommend medical cannabis to patients. Furthermore, participants were asked which sources of information they utilized to know about medical cannabis.

It should be noted that some of the study's results are compared to the "literature" population. A study was published in 2020 about the differences between level of religiosity and students' attitudes toward medical cannabis use (Edelstein et al., 2020a). This study was conducted with 540 students from Ben-Gurion University of the Negev, of whom 196 participants with equivalent characteristics to the studied population, that is nursing students, were used for comparison. Edelstein and colleagues (2020a) studied all the students in the Health Sciences Faculty (nursing, medicine, social work, gerontology). Our study compared the results only to the nursing students from this previous study, 196 out of the total 540.

## Results

### Sample Characteristics

The participants were 221 students. The age range was 18–53 years, with the mean age 24.8 years (*SD* = 5.75). The study population characteristics are described in Table 1.

**Table 1:** Sample characteristics (*N* = 221)

Characteristic	Values	*N*	%
Sex	Women	184	83.3
Marital status	Single	100	45.2
	Living with partner	2	0.9
	Married	112	50.7
	Divorced	7	3.2
Level of religiosity	Secular	8	3.6
	Traditional	8	3.6
	Religious	70	31.7
	Very religious	61	27.6
	Ultra-Orthodox	74	33.5
Studying for degree	B.N	213	96.4
	M.N	8	3.6
Year of study	First	53	24.0
	Second	52	23.5
	Third	59	26.7
	Fourth	57	25.8

### Knowledge and Training

Most of the participants reported feeling insufficiently trained to answer patients' questions about medical cannabis (72.0%). Most of the participants reported having received no formal training regarding medical cannabis (91.0%). Also, most of the participants believed that students in their profession should receive formal training about the use of medical cannabis (98.2%). Most stated that the training should take place both in the classroom and in the clinic/field (59.7%). At the same time, 85.6% of the participants believed nursing students should receive training about the legal aspects of using medical cannabis.

**Fig. 1:**
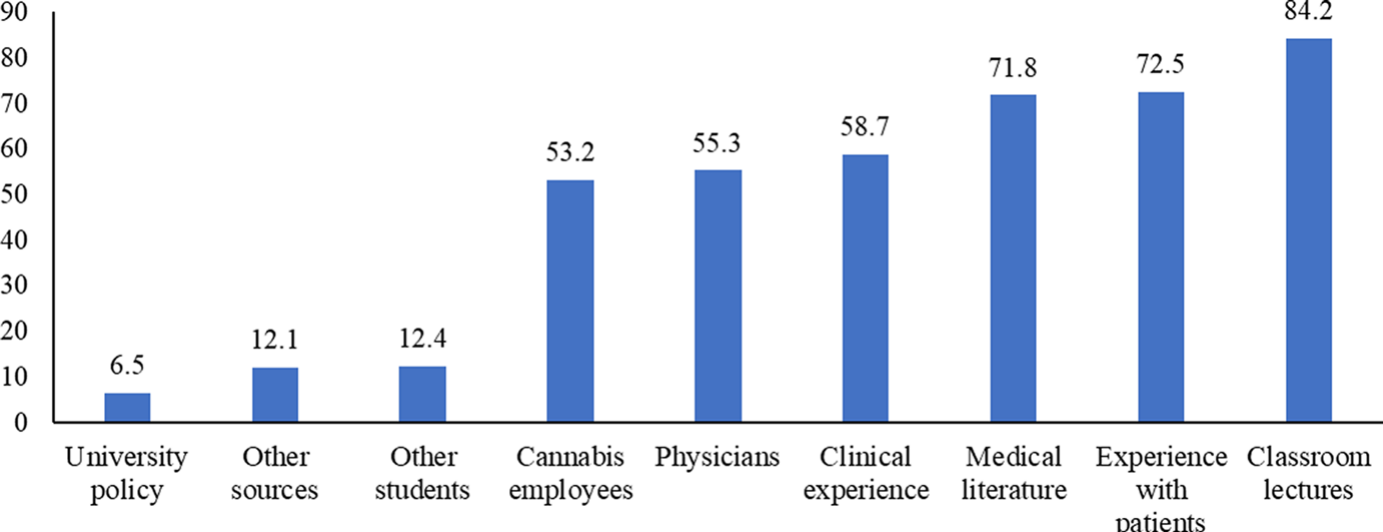
Information sources respondents would use to learn about medical cannabis

The participants were also asked what information sources they would use to learn about medical cannabis (see Fig. 1). The largest percentage would choose to use information from classroom lectures to learn about medical cannabis (84.2%), which the information source perceived as least salient was the university's policy on this issue (6.5%).

### Attitudes and Beliefs

Table 2 shows the distribution of answers about statements examining the attitudes of nursing students from Lev Academic Center and their comparison with nursing students from Ben-Gurion University.

**Table 2:** Distribution of answers on the attitudes and beliefs toward medical cannabis questionnaire

Item	Research population		Nursing students from Ben-Gurion university		*p* value
	(*n* = 221)		(*n* = 196)		
	*n*	%	*n*	%	
I would recommend a patient to use cannabis	181	81.9	183	93.4	< .001
Physicians should recommend cannabis as a medical treatment	188	85.1	187	95.4	< .001
Medical cannabis use has significant health benefits	176	79.6	189	96.4	< .001
Medical cannabis use has significant mental health benefits	176	79.6	172	87.8	.009
Cannabis use involves significant physical health risks	148	67.0	99	50.5	< .001
Cannabis use involves significant mental health risks	152	68.8	123	62.8	.195
Cannabis should be legal for recreational use too	39	17.6	107	54.6	< .001
The government should not declare that cannabis has a high addictive potential ^a^	159	71.9	108	55.1	< .001
The government should not declare that cannabis has no benefit in medical use ^a^	138	62.4	130	66.3	.369
The government should not declare that cannabis is unsafe for use ^a^	127	57.5	106	54.1	.487

Values represent the answer "Agree"

^a^Reversed item. The data are presented after reversal

According to Table 2, the most positive attitude among the current sample was found for the statement: "Physicians should recommend cannabis as a medical treatment" (*n* = 188, 85.1%), whereas among the Ben-Gurion University sample was: “Medical cannabis use has significant health benefits” (*n* = 189, 96.9%).

The lowest agreement among the current sample was with the statement: "Cannabis should be legal for recreational use too" (*n* = 39, 17.6%), while among the Ben-Gurion University sample it was: “The government should not declare that cannabis has no benefit in medical use” (*n* = 65, 33.3%). Nursing students from both institutions think that medical cannabis has significant mental health benefits, and about 6% disagree with this statement.

Most of the study's participants (81.0%) reported that they had never used cannabis recreationally, in contrast with the nursing students at Ben-Gurion University, most of whom are secular, where over half (52.0%) reported having used recreational cannabis during their lifetime.

Most of the students believe that cannabis is addictive, apart from 4.1% who disagree with this statement. This is in contrast with nursing students from Ben-Gurion University, 49.4% of whom disagree that cannabis is addictive.

There were also significant differences between the attitudes of participants with different levels of religiosity regarding medical cannabis (*F*_(220)_ = 1.45, *p* > 0.05). The more religious the students, the more negative their attitude toward medical cannabis. This confirms the research hypothesis. Significant differences also existed between participants who had been exposed to cannabis and those who had not regarding their attitudes to medical cannabis (*t*_(219)_ = 3.38, *p* = 0.001). Participants who had been exposed to medical cannabis expressed more positive attitudes toward medical cannabis.

Also, most students (apart from 3.2%) believed that training about medical cannabis use should be part of the academic curriculum.

### Perceived Efficacy of Cannabis

Table 3 presents the perceived efficacy of cannabis in the current sample.

**Table 3:** Distribution of answers on the perceived efficacy of medical cannabis questionnaire

Medical condition	Effective		Ineffective		Do not know	
	*n*	%	*n*	%	*n*	%
Cancer	195	88.2	12	5.4	14	6.3
Chronic pain	216	97.7	2	0.9	3	1.4
HIV	25	11.3	100	45.2	96	43.4
Chronic intestinal infection	131	59.3	35	15.8	55	24.9
Multiple sclerosis	86	38.9	43	19.5	92	41.6
Parkinson's	96	43.4	55	24.9	70	31.7
Epilepsy/seizures	105	47.5	48	21.7	68	30.8
Terminal illness	186	84.2	11	5.0	24	10.9
Mental disorders (PTSD)	150	67.9	37	16.7	34	15.4

HIV = Human immunodeficiency virus; PTSD = Post traumatic stress disorder

As can be seen from Table 3, most of the participants thought that medical cannabis was effective for treating chronic pain (97.7%), cancer (88.2%), and terminal illness (84.2%). The lowest number of participants thought it was effective for treating HIV, multiple sclerosis, and Parkinson's (11.3%, 38.9%, and 43.4%, respectively).

## Discussion

In recent years, Israel and Western countries have seen a sharp increase in the use of medical cannabis for a range of medical conditions. With the rise in the use of medical cannabis, nurses are at the forefront of care and are available to their patients around the clock. However, the study shows that most of the participants did not feel qualified to answer their patients' questions about medical cannabis (72%). Most (91%) of the students had not received formal training on this topic, and 98.2% believed that nursing students should receive formal training about medical cannabis use.

Also, 85.6% of the participants believed that nursing students should receive training about the legal aspects of medical cannabis use. Evanoff and colleagues (2017) found that medical students who reported receiving training about medical cannabis stated that they were much more willing to answer questions about medical cannabis and to prescribe it to patients than those who had not received training.

It should be noted that when comparing the attitudes of nursing students from Lev Academic Center to those from Ben-Gurion University regarding the need for training about medical cannabis, both populations had similar opinions (97.2% in the study population and 97.4% in Ben-Gurion University) that healthcare and medical staff should receive formal training regarding the use of medical cannabis before recommending it to their patients.

Most of the students supported adding the topic of medical cannabis to the academic curriculum, apart from 3.2% at Lev Academic Center and 4.2% at Ben-Gurion University who did not agree to this unambiguously. Efforts invested in developing a curriculum about medical cannabis will contribute to the accumulation of reliable professional knowledge and to positive attitudes toward the use of cannabis for medical purposes. These results also support the findings of Edelstein (2022) and Findley and colleagues (2021), emphasizing the importance of training.

However, apart from a few private courses, such as a course aimed at qualified professionals, the topic has yet to be included in the academic curriculum as part of the degree studies in any academic institute in Israel and in most places around the world. Despite Israel having medical indications for the use of cannabis published by the Ministry of Health, the students are not familiar with all of them.

One of the main conclusions from a comprehensive meta-analysis by Weisman and Rodriguez (2021) is that it is very important to include the issue of medical cannabis in the curriculum. This is due to the growing recognition of cannabis studies in recent years, both among students and among medical staff. Their strong desire for new programs requires the development and promotion of such curricula, to guarantee evidence-based knowledge and to grant certainty among medical professionals regarding treatment and clinical effects. Likhitsathian and colleagues (2021) also support the importance of education about medical cannabis.

When students were asked about the sources of information they would use to learn about medical cannabis, the largest group of students chose to use information provided in classroom lectures (84.2%). Thus, we can recommend including contents about medical cannabis in classroom lectures, since their wish to receive this information in the classroom supports the conclusion that students want to add this topic to the curriculum.

Despite the limited availability of evidence-based research regarding the risks and benefits of medical cannabis, nursing schools should provide a dedicated curriculum and promote a professional discourse aimed at allowing nursing students to interpret correctly the existing information and to ensure appropriate care for patients (Zolotov et al., 2021a).

Regarding students' attitudes, the study's findings show that the more religious the students, the more negative their attitudes toward medical cannabis. This aligns with other research on this topic, showing that the attitude of religious students toward the use of cannabis for medical purposes and toward legalization of recreational use were more negative (Edelstein et al., 2020a, 2020b). This can be explained by the different lifestyle of the ultra-Orthodox community, which is opposed to modern society and very different to non-ultra-Orthodox life (Bilu & Witztum, 1993).

Most of the ultra-Orthodox live in closed communities and send their children to religious Jewish schools. Their exposure to the media is limited; television, cinema, theater, reading literature, and using the internet are very limited and strictly controlled (Loewenthal, 2014). Religion serves as an important shield against the use of alcohol and other drugs (Wallace, 2003). There is extensive evidence that rabbis' rulings and instructions have a large following, which may partially explain the lower incidence of addiction among ultra-Orthodox Jews (Loewenthal, 2014). Religious leaders play a crucial role in guiding health decisions, balancing traditional beliefs with modern medical practices (Woolford & Horner, 2024).

Regarding the religious population as a whole, it has been found that a religious environment and religious education are linked to lower alcohol use and risky behavior (Isralowitz & Reznik, 2015). Since cannabis is currently defined as a drug in Israel, there is less use of it among the religious population.

According to Finkelstein (2017), the only explained reference by the great rabbis in Israel to using cannabis products is a short reply by Rabbi Moshe Feinstein, written in 1973, where he forbids the smoking of cannabis for various reasons, such as: physical and cognitive damage, debauchery, removal of restraint, and disrespect of parents. Because in the past, and sometimes also in the present, the use of cannabis was not identified with normative behavior but was perceived as part of a criminal culture, this rabbinical and institutional opposition is understandable.

The strict attitude toward cannabis products among most rabbis led to the ban on the use of cannabis. The ruling mentioned above probably influenced the attitudes of religious students toward the use of cannabis. However, in an article from 2016, Rabbi Reznikov permitted the use of cannabis *for medical purposes*. The rabbi did not view it as a drug but as a medicine that relieves suffering, and he even permitted its use on the Sabbath according to Halakhic priority order: food, pills, steaming, and smoking.

However, he understood that one of the problems is that cannabis use for recreational purposes is popular, and that there is a leaking of cannabis into the "black market", for non-medical purposes, which he does not support (Reznikov, 2016). His attitude is reflected in the study's findings showing that the participants recognize the medical benefits of cannabis but oppose its recreational use.

The study found that the most positive attitude among the students was that "The use of medical cannabis has significant health benefits" (mean 1.57), and the fewest participants believed that "Cannabis should be legal for recreational purposes too" (mean 2.66). Like Rabbi Reznikov, the students supported the use of *medical* cannabis and believed that it could have great benefits for medical conditions, but they opposed the recreational use of cannabis.

If we compare the study's findings with Isralowitz and colleagues (2021), less than a fifth of nursing students at Lev Academic Center think that cannabis should be legal for recreational purposes too, compared with over half the healthcare professions students at Ben-Gurion University. According to researchers from Ben-Gurion University, this could be partially linked to the fact that the Israeli media greatly supports the benefits of medical cannabis, and such reports could increase the positive attitude toward its use (Isralowitz et al., 2021).

In contrast, at the Lev Academic Center the student population is more conservative and perhaps less exposed to the national media, but instead only to conservative media that report and stress completely different contents. The more religious the students and the more closed their society, the less exposed they are to national media. This could explain their more negative attitudes, compared to traditional students, for example. This demonstrates the need to teach the topic in the classroom, to present up-to-date studies, and to show objective data about medical cannabis, particularly for religious student populations. A recent study conducted among Orthodox Jewish university and college students in the USA also concluded that higher levels of religiosity correlate negatively with cannabis use (Liberman, 2024).

Another explanation for the finding that most of the study's participants disagree with the legalization of cannabis for recreational purposes is that most students believe cannabis is addictive, apart from 4.1% who disagree with this statement. This is in contrast with the nursing students at Ben-Gurion University. Forty-nine-point four percent of the students there reported that they did not agree that cannabis was addictive. This comparison shows once again the gap between the attitudes of secular students compared with religious students who have a more conservative lifestyle. However, despite religious students believing that cannabis is addictive, they support its *medical* use.

Most of the study's participants (81%) reported that they had never used cannabis recreationally, unlike the mostly secular nursing students at Ben-Gurion University, over half of whom (52%) reported that they had used cannabis recreationally during their lifetime. According to the researchers, people who use cannabis or know cannabis users personally are more likely to hold positive or permissive attitudes toward cannabis (Isralowitz et al., 2021). Regarding students who had used cannabis in the past, researchers assume that their personal experience of not having any negative effects after using cannabis convinced them that the historical attitude to the risks of medical cannabis was exaggerated (Chan et al., 2017).

## Study Limitations

The study was conducted among nursing students in one academic institution, where medical cannabis is not included in the degree curriculum. Also, this was a cross-sectional study based on a relatively small sample (221 students). The sample does not fully reflect the levels of religiosity in the population, did not exclude non-religious students, as it contains eight secular students and eight traditional students, so the ability to generalize the study's findings to other populations of religious/ultra-Orthodox nursing students is limited. Also, the number of men in the sample was low.

## Conclusions

There is a mismatch between the legalization of medical cannabis in Israel and the lack of preparation of healthcare professions students, particularly nursing students, to work with its users. The number of permits and medical indications is constantly rising, and expected to continue rising, and so there must be a change in the preparation of students to the new reality. It is necessary to develop curricula, to include the topic of medical cannabis in the training, and to invest in training the next generation of nursing students, particularly from the religious and ultra-Orthodox sector, so that the knowledge they acquire is formal, valid, and reliable, and so that it will influence their attitudes.

A follow-up study could be conducted to examine attitudes and knowledge among additional populations: nursing department teachers, nursing and healthcare professionals, or cannabis users themselves. Perhaps medical cannabis users can be compared with recreational cannabis users. Staff members who had taken courses on medical cannabis and those who had not studied the topic could be compared to test how much courses about medical cannabis create knowledge and influence attitudes.

Finally, an in-depth study could include interviews with policy makers, deans, and department heads, to clarify their attitudes to examine whether they are considering including the topic of medical cannabis in the curriculum, and how this can be achieved.
